# Multivariable Regression Analysis of Clinical Data from the Randomized-Controlled EffPac Trial: Efficacy of Femoropopliteal Drug-Coated Balloon Angioplasty

**DOI:** 10.1007/s00270-020-02452-2

**Published:** 2020-04-01

**Authors:** Selma Mietz, Thomas Lehmann, Ulf Teichgräber

**Affiliations:** 1 Department of Radiology, Jena University Hospital, Jena, Germany; 2 Center for Clinical Studies, Jena University Hospital, Jena, Germany; 3 Institut für Diagnostische und Interventionelle Radiologie, Universitätsklinikum Jena, Am Klinikum 1, 07747 Jena, Germany

**Keywords:** Drug-coated balloon angioplasty, Femoropopliteal lesions, Hypertension, Regression analysis

## Abstract

**Purpose:**

The post-hoc multivariable analysis of EffPac study data aimed to identify explanatory variables for efficacy of femoropopliteal artery angioplasty.

**Methods:**

In the prospective, randomized, controlled EffPac study, patients were allocated to either DCB or plain old balloon angioplasty. Multivariable regression including interaction analysis was conducted to assess the impact of selected variables on the outcome measures of late lumen loss (LLL) at 6 months, and on binary restenosis, target lesion revascularization (TLR), clinical improvement, and hemodynamic improvement at 12 months.

**Results:**

A total of 171 patients (69 ± 8 years, 111 men) were treated at 11 German centers. Hypertension increased, and advanced age decreased LLL (*B* coefficient [*B*]: 0.7 [95% CI − 0.04 to 1.3], *p* = 0.06 and − 0.3 per 10 years [95% CI − 0.5 to 0.01], *p* = 0.06, respectively). DCB angioplasty decreased odds of 12-month TLR and binary restenosis (OR 0.4 [95% CI 0.2 to 0.8], *p* = 0.01 and OR 0.1 [95% CI 0.01 to 0.6], *p* = 0.02, respectively). Lesion length and severe calcification decreased clinical improvement (*B*: − 0.1 per 10 mm [95% CI − 0.1 to − 0.03], *p* = 0.001 and − 0.1 [95% CI − 1.7 to − 0.1], *p* = 0.03, respectively). DCB angioplasty in former smokers improved ABI (0.2 [95% CI 0.01 to 0.5], *p* = 0.04).

**Conclusion:**

DCB angioplasty decreased the incidence of 12-month restenosis and TLR. Increasing lesion length and severe calcification reduced clinical improvement. Hypertension is suspected to facilitate, and advanced age to mitigate LLL. DCB improved ABI most in former smokers.

**Electronic supplementary material:**

The online version of this article (10.1007/s00270-020-02452-2) contains supplementary material, which is available to authorized users.

## Introduction

Numerous randomized trials on femoropopliteal artery disease demonstrated that drug-coated balloon (DCB) angioplasty effectively decreased late lumen loss (LLL) [1–9] and increased primary patency [10–14]. However, heterogeneity among studies is substantial: The mean number of patients needed to treat within 1 year to prevent a single target lesion revascularization (TLR) ranges between 4 and 33 [15].

 Different effect sizes of DCB across studies mainly originate from DCB types or study-specific procedure details, whereas effect size within studies may depend on patient and lesion characteristics. For instance, Albrecht et al. reported on greater LLL and Laird et al. found a lower incidence of primary patency with increasing lesion length after both DCB and plain old balloon angioplasty (POBA) [16, 17]. Scheinert et al. found lesion length, total occlusion, and critical limb ischemia to be associated with 12-month TLR after DCB [18]. Fanelli et al. described a worse impact of severe calcification on LLL and primary patency after DCB angioplasty [19], and severe dissections were identified as risk factor for restenosis after POBA [20, 21].

The randomized EffPac trial revealed a smaller 6-month LLL (primary endpoint), a higher incidence of 12-month primary patency, and a lower risk of 12-month TLR after DCB angioplasty compared to POBA [22]. The purpose of this post-hoc interaction and multivariable analysis of EffPac trial data was to assess interaction effects of selected explanatory variables with treatment, and to identify predictors of morphologic, clinical, and hemodynamic outcome measures after femoropopliteal angioplasty.

## Methods

### Study Design, Patients, and Procedure

Consecutive patients with symptomatic, atherosclerotic, femoropopliteal stenosis, or occlusion ≤ 150 mm were included in the prospective, randomized, controlled trial at 11 German sites between September 2015 and December 2016. Patients were allocated 1:1 to either DCB or POBA and blinded to the treatment strategy. Successful pre-dilation for at least 30 s without flow-limiting dissection or relevant residual stenosis was a precondition to inclusion. Detailed eligibility criteria are available (online resource 1, Table A1).

Investigators were not blinded to the study device. The investigational DCB (Luminor-35 paclitaxel-coated balloon, iVascular S.L.U., Barcelona, Spain) was used according to manufacturer’s instruction. Inflation of DCB or control device (standard balloon) should have lasted 60 ± 10 s up to the nominal pressure. Bailout stenting in case of flow-limiting dissection despite prolonged inflation was permitted. The angiographic and Doppler sonographic results were evaluated by an independent core laboratory.

Detailed description of device and procedure, and the full study protocol had been provided earlier [22, 23]. The EffPac trial is registered with ClinicalTrials.gov (NCT02540018).

### Study Outcome Measures

Primary endpoint of the EffPac trial was 6-month LLL. Outcomes measured of this post-hoc analysis were impact of baseline patient, lesion, and procedure characteristics on 6-month LLL, binary restenosis, TLR, improvement in Rutherford–Becker category (clinical improvement), and improvement in ankle-brachial index (ABI) (hemodynamic improvement) at 12 months. Outcome measures were specified as unstandardized regression coefficient *B* (*B*) in case of LLL, Rutherford–Becker category, and ABI or as odds ratio (OR) in case of binary restenosis and TLR. LLL was defined as difference between minimum lumen diameter immediately after angioplasty and at follow-up. Restenosis occurred with > 50% diameter stenosis of the target lesion by angiography or peak systolic velocity ratio of ≥ 2.5 by duplex ultrasound. Detailed description of outcome measures is available at online resource 1.

### Statistical Analysis

Post-hoc multivariable regression analysis was conducted in three steps separately for each outcome measure. In the first step, 15 independent variables (age, sex, hypertension, body mass index, diabetes mellitus, smoking status, percentage diameter stenosis, lesion length, calcification, total occlusion, bailout stenting, dissection, Rutherford–Becker category, ABI, Transatlantic Inter-Society Consensus for the Management of Peripheral Arterial Disease classification (TASC) II classification) were subjected to formal interaction analysis with treatment. In case of a *p* value for interaction of < 0.1, variables concerned, and their related interaction terms were included in the final multivariable analysis (online resource 1, Table A2). In the second step, variables that showed a *p* value for interaction of ≥ 0.1 were subjected to univariable analysis (linear or logistic regression). Variables with a *p* value of < 0.1 for univariable association with the respective outcome were included in the final multivariable analysis (online resource 1, Table A3). In the third step, all variables with *p* < 0.1 at step 1 or 2, and the variable of treatment were included in the final model. Additionally, box plots and Spearman’s correlation were provided to visualize descriptive associations of clinically relevant variables to LLL (lesions with bailout stenting were excluded) and hemodynamic improvement. Continuous variables were expressed as mean ± standard deviation, and differences were assessed by Mann–Whitney U test. Categorical variables were reported as count and percentage, and differences were assessed by Fisher’s exact test. Statistical analysis was performed using SPSS Statistics 25.0 (IBM, Armonk, NY, USA).

## Results

### Study Population

Baseline patient, lesion, and procedure characteristics were well matched across treatment groups (Table 1). Six-month LLL was assessed in 53 of 85 (62.4%) DCB and in 63 of 86 (73.3%) POBA patients. One-year follow-up was completed in 76 (89.4%) DCB and 76 (88.4%) POBA patients. Outcomes at 6 and 12 months were published previously [22]. In short, DCB proved to be effective regarding 6-month LLL, 12-month primary patency, and TLR.

**Table 1:** Patient, lesion, and procedure characteristics

Characteristics	Total (*n* = 171)	DCB (*n* = 85)	POBA (*n* = 86)	*p* value
Age, year	68.1 ± 8.2	68.0 ± 7.5	68.1 ± 8.8	*p* = 0.96
Male	111 (64.9)	51 (60.0)	60 (69.8)	*p* = 0.20
Body mass index^a^	27.5 ± 4.7	27.4 ± 4.8	27.7 ± 4.7	*p* = 0.57
Underweight	4 (2.4)	1 (1.2)	3 (3.6)	
Normal weight	50 (30.5)	30 (37.0)	20 (24.1)	
Pre-obesity	68 (41.5)	28 (34.6)	40 (48.2)	
Obesity	42 (25.6)	22 (27.2)	20 (24.1)	
Smoking status				*p* = 0.94
Current smoker	71 (41.8)	34 (40.5)	37 (43.0)	
Former smoker	71 (41.8)	36 (42.9)	35 (40.7)	
Never smoked	28 (16.5)	14 (16.7)	14 (16.3)	
Diabetes mellitus	66 (38.6)	31 (36.5)	35 (40.7)	*p* = 0.64
Hypertension	147 (86.0)	74 (87.1)	73 (84.9)	*p* = 0.83
Hyperlipidemia	119 (69.6)	60 (70.6)	59 (68.6)	*p* > 0.99
Rutherford category				*p* = 0.53
2	31 (18.2)	13 (15.3)	18 (21.2)	
3	135 (79.4)	69 (81.2)	66 (77.6)	
4	3 (1.8)	2 (2.4)	1 (1.2)	
5	1 (0.6)	1 (1.2)	0	
Ankle-brachial index	0.74 ± 0.23	0.73 ± 0.23	0.74 ± 0.23	*p* = 0.78
Lesion length, mm	57.4 ± 41.2	59.1 ± 43.4	55.8 ± 39.1	*p* = 0.60
Total occlusion	39 (22.9)	17 (20.2)	22 (25.6)	*p* = 0.47
Diameter stenosis, %	89.1 ± 9.3	88.0 ± 9.8	90.1 ± 8.8	*p* = 0.16
TASC II^b^				*p* = 0.75
A	113 (66.1)	55 (64.7)	58 (67.4)	
B	58 (33.9)	30 (35.3)	28 (32.6)	
Calcification				*p* = 0.11
None/mild	83 (49.1)	45 (54.2)	38 (44.2)	
Moderate	73 (43.2)	35 (42.2)	38 (44.2)	
Severe	13 (7.7)	3 (3.6)	10 (11.6)	
Dissection	67 (39.2)	32 (37.6)	35 (40.7)	*p* = 0.76
Bailout stenting	29 (17.1)	13 (15.3)	16 (18.8)	*p* = 0.68

Categorical values are presented as counts (percentages); continuous values are presented as mean ± standard deviation

*DCB* drug-coated balloon, *POBA* plain old balloon angioplasty

^a^Body mass index (kg/m^2^) according to the World Health Organization (WHO) definition: normal weight 18.5–24.9, pre-obesity 25.0–29.9, obesity ≥ 30

^b^Inter-Society Consensus for the Management of Peripheral Arterial Disease classification

### Predictors of Morphologic Outcome Measures

#### Late Lumen Loss

**Fig. 1:**
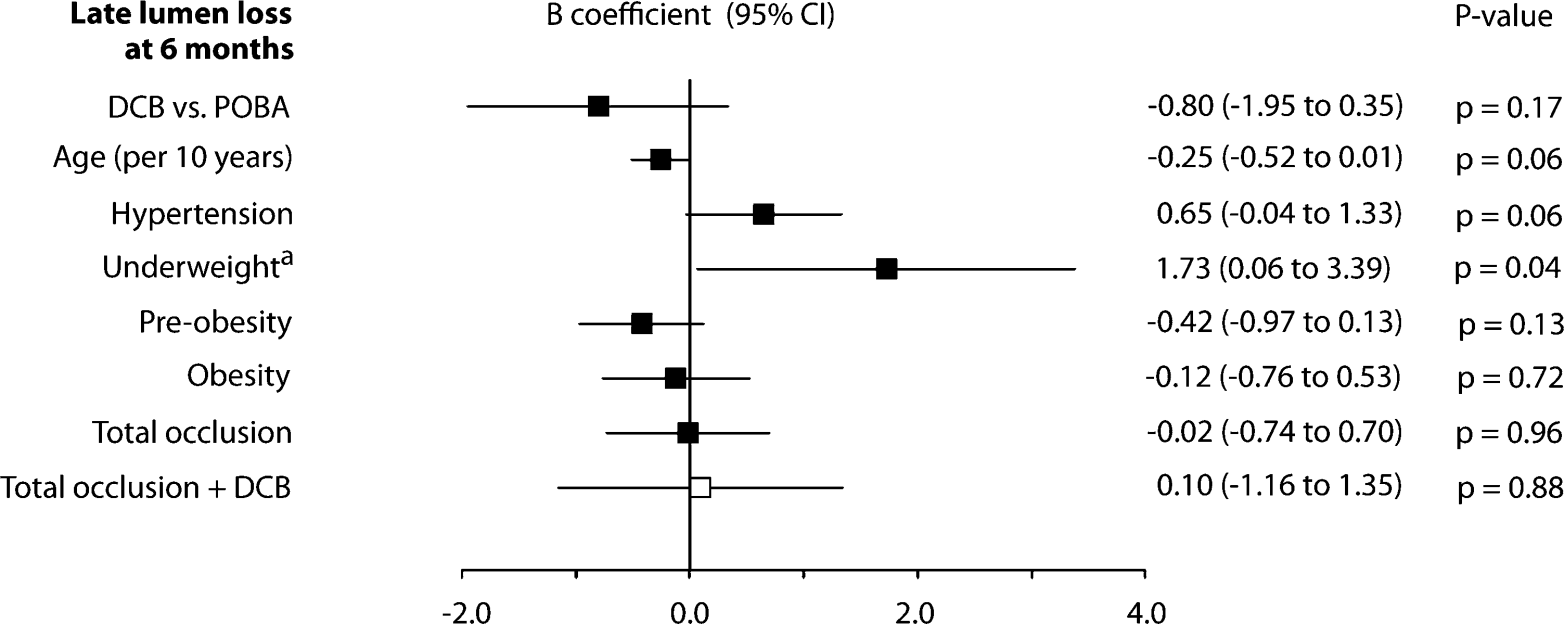
Association of late lumen loss at 6 months with selected baseline and procedure variables, determined by post-hoc multivariable analysis. *B* coefficients of single independent variables are pictured as black squares and joint effects of independent variables with treatment as white squares with their corresponding 95% confidence intervals. ^a^Four underweight patients were analyzed. *DCB* drug-coated balloon, *POBA* plain old balloon angioplasty, *TASC* Inter-Society Consensus for the Management of Peripheral Arterial Disease classification

For the joint effect of CTO and treatment on LLL, a *p* value of 0.06 was determined, and thus, according to study protocol, CTO and its corresponding interaction term were included in the multivariable model (online resource 1, Table A2). Finally, multivariable analysis did not reveal a simultaneous impact of CTO and treatment on LLL (Fig. 1).

**Fig. 2:**
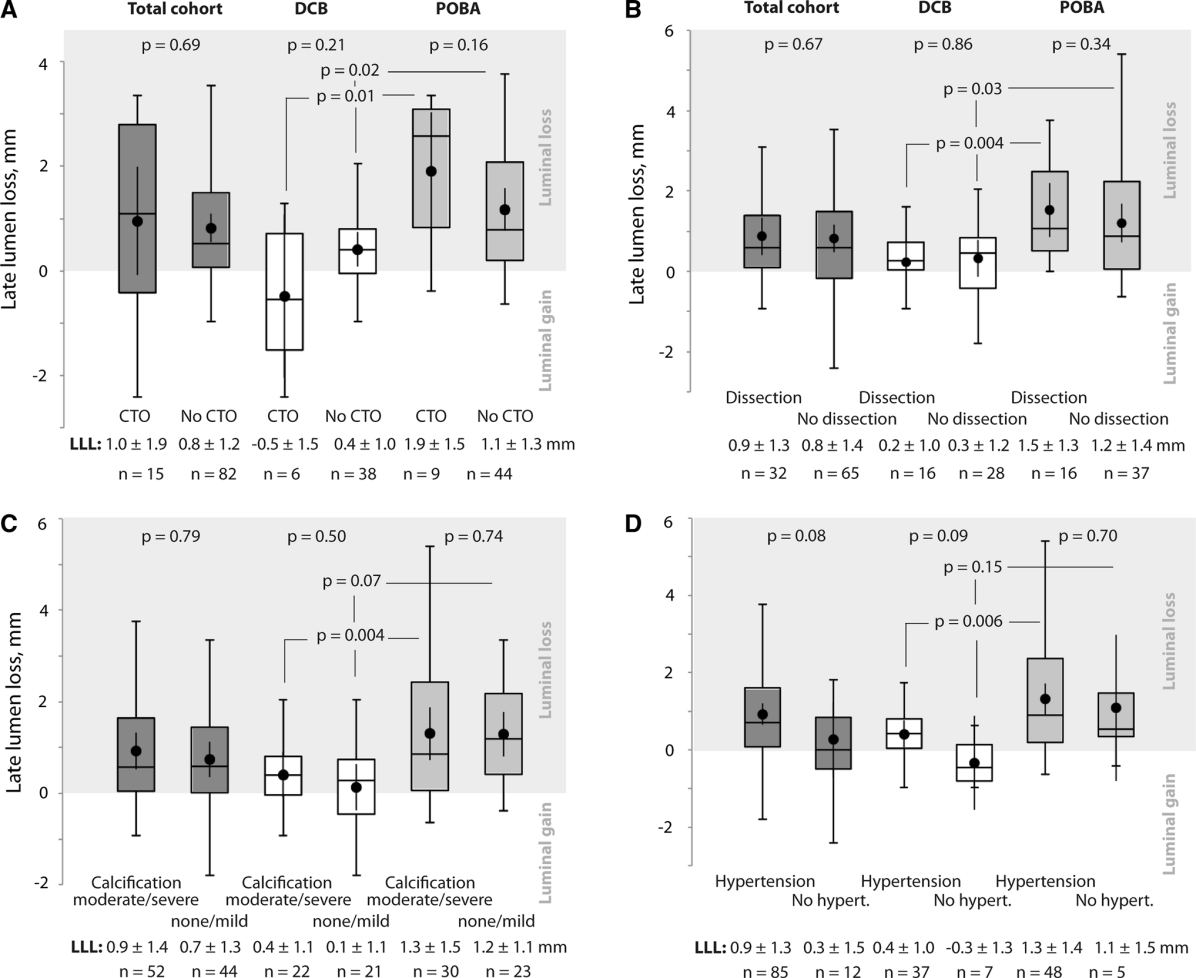
Association of 6-month late lumen loss with total occlusion (**A**), dissection (**B**), moderate or severe calcification (**C**), and hypertension (**D**). Lesions that were treated with stent were excluded from this analysis. Box plots indicate median and interquartile ranges. Whiskers end with the lowest and highest data point within 1.5 × IQR. Dots represent means with their corresponding 95% confidence interval. *CTO* chronic total occlusion, *DCB* drug-coated balloon, *LLL* late lumen loss, *POBA* plain old balloon angioplasty

*Treatment* Multivariable regression revealed a trend to decrease LLL with DCB compared to POBA (*B* coefficient [*B*]: − 0.8 [95% CI − 2.0 to 0.4], *p* = 0.17) (Fig. 1A). This finding was supported by results from descriptive statistics that found less LLL after DCB versus POBA irrespective of whether or not arteries were occluded, dissected, moderately or severely calcified, or whether patients had hypertension (Fig. 2A–D).

**Fig. 3:**
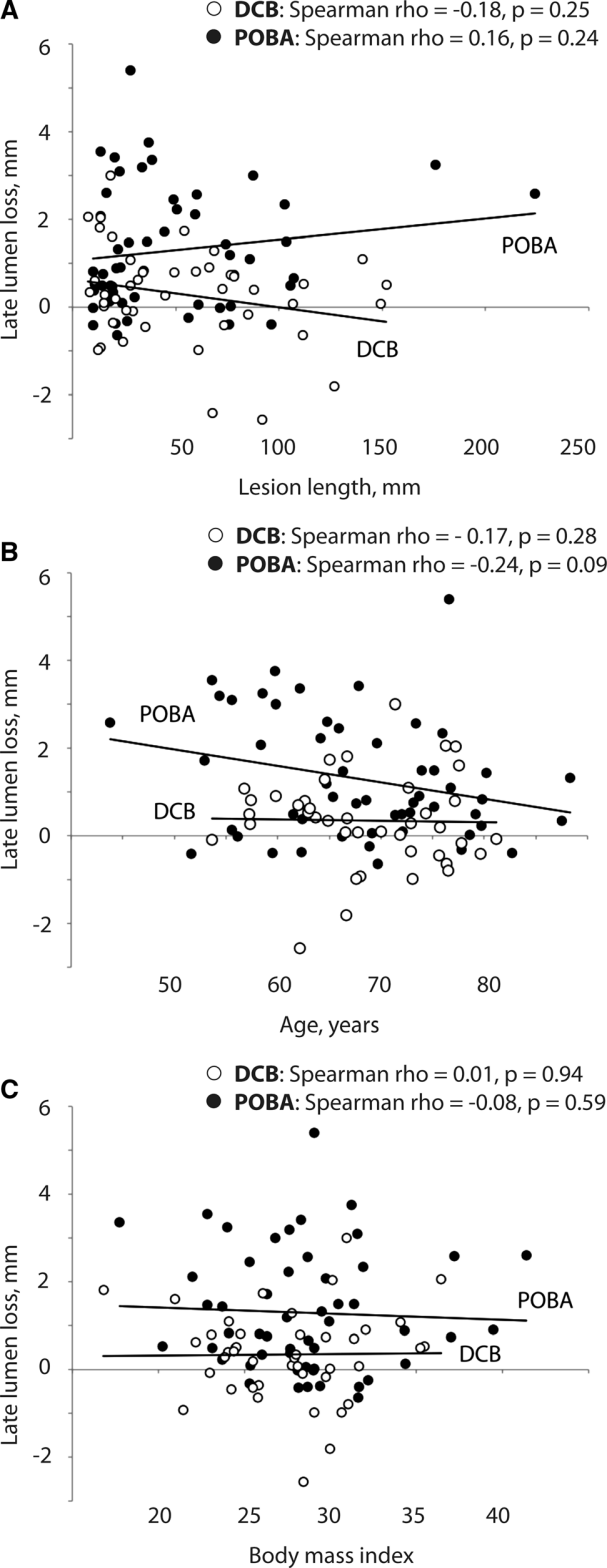
Association of 6-month late lumen loss with lesion length (**A**), age (**B**), and body mass index (**C**) Lesions that were treated with stent were excluded from this analysis. *DCB* drug-coated balloon, *POBA* plain old balloon angioplasty

*Lesion complexity* Total occlusion, dissection, and calcification did not affect LLL in both treatment groups (Fig. 2A–C). However, with DCB there was a weak negative correlation between LLL and **l**esion length (DCB: *r*_*s*_ = − 0.18, *p* = 0.25; POBA: *r*_*s*_ = 0.16, *p* = 0.24). Therefore, benefit of DCB tended to rise with increasing lesion length (Fig. 3A).

*Hypertension* Hypertension tended to increase LLL (*B*: 0.7 [95% CI − 0.04 to 1.3], *p* = 0.06) (Fig. 1). The impact was more apparent in the DCB than in the POBA group (0.4 vs. − 0.3 mm, *p* = 0.09 and 1.3 vs. 1.1 mm, *p* = 0.70, respectively) (Fig. 2D).

*Age* Higher age tended to decrease LLL (*B*: − 0.3 [95% CI − 0.5 to 0.01], *p* = 0.06) (Fig. 1A). A weak negative correlation was visible with POBA (r_s_ = − 0.24, *p* = 0.09). Thus, the benefit of DCB over POBA tended to decrease with advanced age (Fig. 3B).

*Body mass index* No correlation was seen between LLL and BMI (*r*_*s*_ = 0.01, *p* = 0.94 with DCB, *r*_*s*_ = 0.08, *p* = 0.59 with POBA) (Fig. 3C).

#### Binary Restenosis

**Fig. 4:**
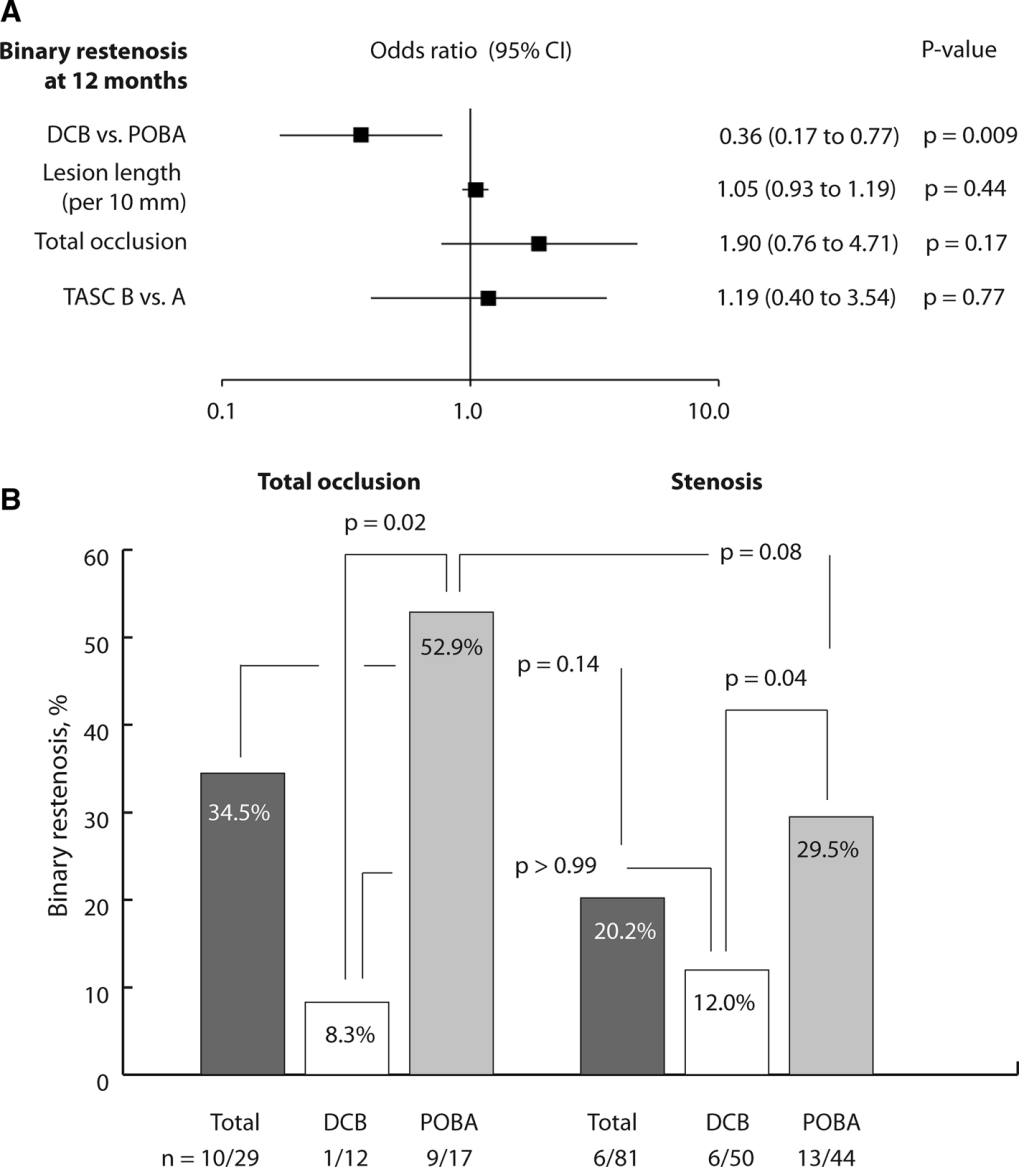
Association of 12-month binary restenosis with selected baseline and procedure variables, determined by post-hoc multivariable analysis (**A**), and association of 12-month binary restenosis with total occlusion, excluding bailout stenting (**B**). *DCB* drug-coated balloon, *POBA* plain old balloon angioplasty, *TASC* Inter-Society Consensus for the Management of Peripheral Arterial Disease classification

Six-month luminal gain was associated with less binary restenosis than luminal loss (0% vs. 33.8%, *p* = 0.001, Table 2). None of the selected variables interacted with the treatment effect on binary restenosis (online resource 1, Table A2). Multivariable analysis revealed that DCB angioplasty independently reduced the odds of binary restenosis (OR 0.4 [95% CI 0.2 to 0.8], *p* = 0.01) (Fig. 4A).

**Table 2:** Association of late luminal loss at 6 months with morphologic and clinical outcomes at 12 months

Outcomes	Luminal loss at 6 months	Luminal gain at 6 months	*p* value
Binary restenosis	24/71 (33.8)	0/23 (0.0)	*p* = 0.001
Target lesion revascularization	12/65 (18.5)	0/21 (0.0)	*p* = 0.03

Values are given as counts (percentages)

Patients who underwent bailout stenting were excluded from analysis

In the POBA, but not in the DCB group, total occlusions tended to be more frequently restenosed (POBA: 52.9% vs. 29.5%, *p* = 0.08 vs. DCB: 8.3% vs. 12.0%, *p* > 0.99) (Fig. 4B).

### Predictors of Clinical Outcome Measures

**Fig. 5:**
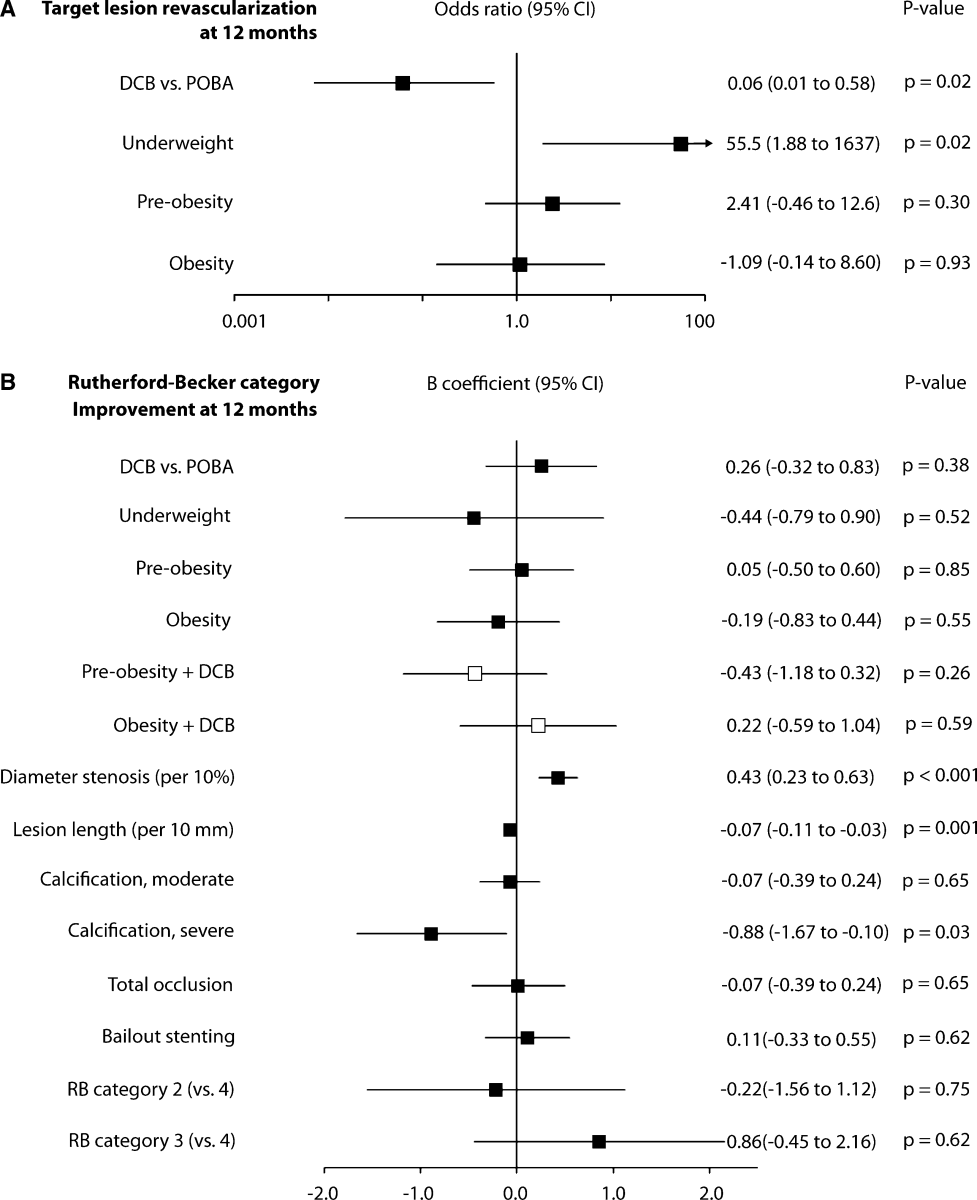
Association of target lesion revascularization (**A**) and improvement in Rutherford–Becker category (**B**) at 12 months with selected baseline and procedure variables, determined by post-hoc multivariable analysis. *B* coefficients of single independent variables are pictured as black squares and joint effects of independent variables with treatment as white squares with their corresponding 95% confidence intervals. *DCB* drug-coated balloon, *POBA* plain old balloon angioplasty, *RB* Rutherford–Becker category

*Target lesion revascularization* Luminal gain at 6 months was associated with less 12-month TLR than luminal loss (0% vs. 18.5%, *p* = 0.03, Table 2). None of the selected variables interacted with the treatment effect (online resource 1, Table A2). Multivariable analysis found TLR to be less likely in patients who underwent DCB angioplasty (OR 0.1 [95% CI 0.01 to 0.6], *p* = 0.02) (Fig. 5A).

*Clinical improvement* Interaction analysis of nutritional status with treatment revealed a *p* value of 0.03 (online resource 1, Table A2), and thus, nutritional status and its corresponding interaction term were included in the final multivariable analysis. Finally, combined effect of nutritional status and treatment did not impact clinical improvement. The same applied for the variable of treatment alone, that did not affect clinical improvement (*B*: 0.3 [95% CI − 0.3 to 0.8], *p* = 0.38). However, it should be noted that patients who underwent TLR were included in the assessment of Rutherford–Becker category at 12 months. Lesion length and severe calcification turned out to independently decrease clinical improvement (*B*: − 0.07 per 10 mm [95% CI − 0.1 to − 0.03], *p* = 0.001 and *B*: − 0.88 [95% CI − 1.7 to − 0.1], *p* = 0.03, respectively). Lesions that were more severely stenosed at baseline were associated with increased clinical improvement (Fig. 5B).

### Predictors of Hemodynamic Improvement

**Fig. 6:**
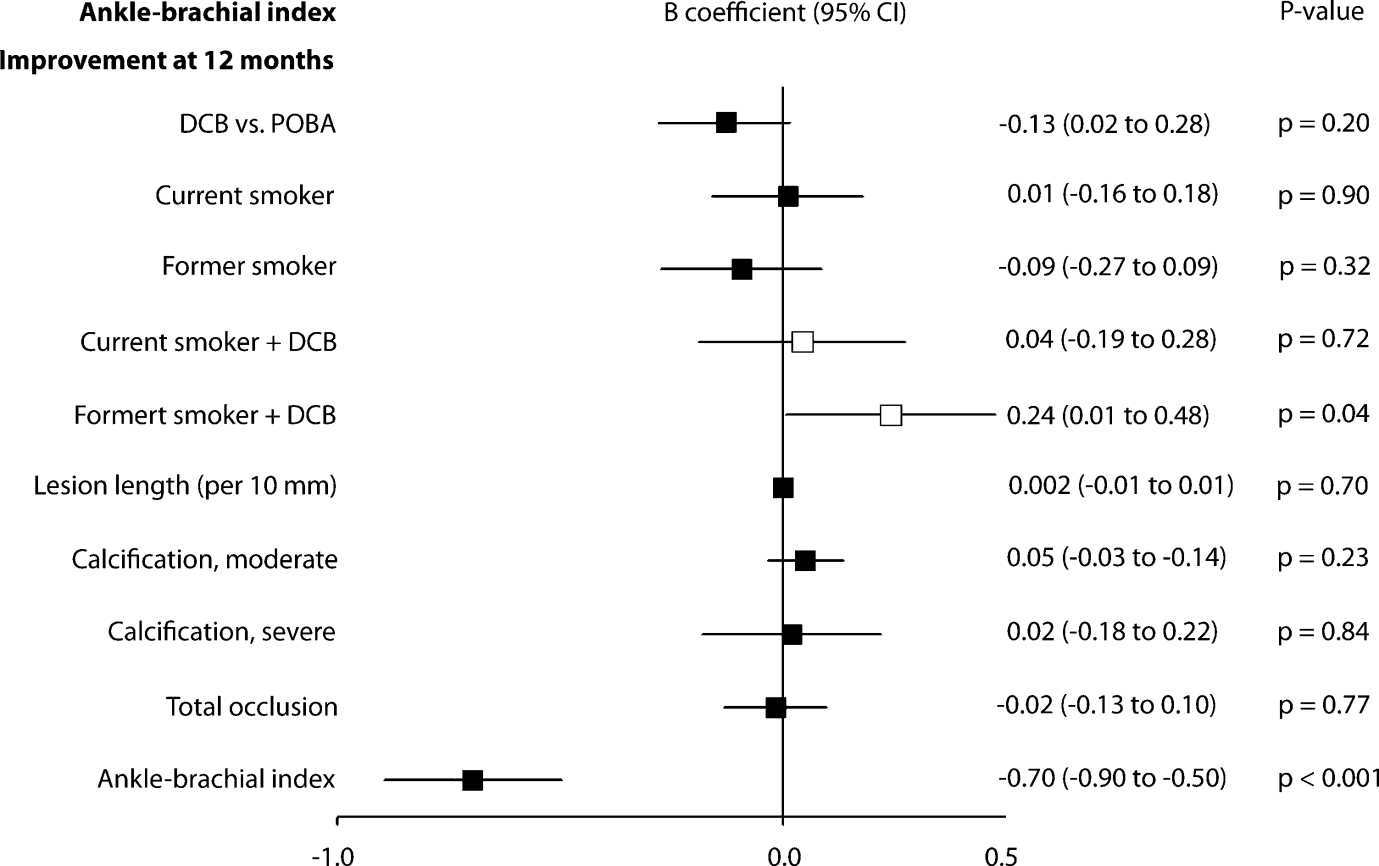
Association of improvement in ankle-brachial index (ABI) at 12 months with selected baseline and procedure variables, determined by post-hoc multivariable analysis. *B* coefficients of single independent variables are pictured as black squares and joint effects of independent variables with treatment as white squares with their corresponding 95% confidence intervals. *DCB* drug-coated balloon, *POBA* plain old balloon angioplasty, *TASC* Inter-Society Consensus for the Management of Peripheral Arterial Disease classification

Simultaneous effect of smoking status and treatment on hemodynamic improvement revealed a *p* value of 0.02. Thus, smoking status and related interaction terms were included in the multivariable analysis (online resource 1, Table A2). Multivariable analysis indicated that DCB angioplasty significantly improved hemodynamic condition in former smokers (*B*: 0.2 [95% CI 0.01 to 0.48], *p* = 0.04). ABI improvement did not differ between treatment groups in general (DCB angioplasty vs. POBA: *B*: − 0.13 [95% CI 0.02 to 0.28], *p* = 0.20). However, patients who underwent TLR were included in 12-month ABI assessment. A higher ABI at baseline was associated with less clinical improvement (Fig. 6).

## Discussion

DCB proved to be superior to POBA in both, prevention of restenosis and TLR. Lesion length and severe calcification were identified as predictors of less clinical improvement. DCB angioplasty decreased 6-month LLL more effectively than POBA, irrespective of whether or not arteries were occluded, calcified, or dissected. Our study revealed a series of hypothesis generating results on LLL. First, increasing lesion length tended to increase the benefit of DCB over POBA; second, hypertension tended to increase LLL, and third, advanced age tended to decrease LLL. No correlation was seen between LLL and BMI. Former smoker emerged as predictors of increased hemodynamic improvement after DCB. Sex, diabetes, TASC II classification, and bailout stenting did not predict outcomes.

### Complex Lesions

In contrast to previous studies, in our study, increasing lesion length was not correlated with LLL [16–18]. However, the outcome of LLL only describes the quantity of loss in diameter, but not the total length of relevant luminal loss. Thus, even with similar LLL, longer lesions may have worse outcomes with respect to binary restenosis or clinical improvement, as observed in this study. The tendency of growing benefit of DCB with increasing lesion length was possibly due to chance. A dose–response relationship with paclitaxel in longer lesions might be considered.

The slight tendency of increased 6-month LLL in total occlusions treated with POBA is encouraged by findings on 12-month binary restenosis. Although calcium is suspected to impede paclitaxel penetration into the vessel wall [19], our study could not detect any disadvantage of moderately or severely calcified lesions regarding LLL in the DCB group. This may be explained by the assessment of calcification that was done only by visual estimate. Another explanation might be that in case of severe calcification, pre-dilation sufficiently loosened the calcium barrier. Finally, investigators might have positioned DCB not directly at circumferentially calcified segments of the lesion. Additional stent implantation might have supported balloon angioplasty. Despite no impact of calcification on LLL, severe calcification predicted less clinical improvement. This might be attributable to more advanced stages of disease and involvement of infrapopliteal arteries in patients with severe calcification.

Dissected lesions that were not stented did not differ in the magnitude of LLL from not dissected lesions in both treatment groups. Superiority of DCB over POBA was proven for both, dissected and not dissected lesions. However, stented dissections were excluded from analysis on LLL. Thus, our finding does not contradict previous studies that reported on increased restenosis rates after severe, but not after mild dissection [20, 21]. In our study, neither dissection nor bailout stenting predicted restenosis.

Clinical improvement increased with higher percentage diameter stenosis, and hemodynamic improvement decreased with higher baseline ABI. This might be attributable to more room for improvement by revascularization in lesions with poor baseline conditions, and vice versa.

### Comorbidities

The trend of decreased LLL with advanced age in the POBA group is not supported by previous studies and possibly due to chance [16, 17]. However, Han et al. reported on reduced neointimal thickness and strut coverage of coronary arteries after stent implantation in older patients [24]. This might have been due to declined endothelial function and regenerative capacity. In elderlies, synthesis of growth factors is decreased, and growth factor receptors are suppressed. This leads to an attenuated response to physical and chemical signals [25–27].

The observed tendency of an unfavorable impact of hypertension on LLL might have been due to chance. Only a few patients without hypertension were included in the analysis, and no information on control of hypertension or medication was available. Patients without a history of hypertension might have benefited from sufficient endothelial function.

Regarding ABI, former smokers benefited most from DCB angioplasty compared to never or current smokers. Smoking induces inflammation and modulates immune response [28]. Possibly, former smokers responded well to paclitaxel because of a still altered inflammation response from previous smoking together with an already restored vascular function from smoking cessation.

### Limitations

Our study was a post-hoc evaluation of prospectively achieved EffPac trial data. The EffPac trial was designated neither to investigate associations of explanatory variables with clinical outcomes nor to determine interaction effects. Thus, power and evidence are limited and findings on age, hypertension, and smoking status should be considered hypothesis generating. Study device was the Luminor-35 DCB, and thus, results may not apply equally for all DCB types.

## Conclusion

Treatment strategy of DCB or POBA in femoropopliteal lesions proved to independently affect incidence of restenosis and TLR. Sex, diabetes, total occlusion, dissection, bailout stenting, baseline Rutherford category, and TASC II classification were not found to impact outcome measures. Increased lesion length and severe calcification independently decreased clinical improvement. In addition, our study revealed a trend for age to decrease and for hypertension to increase LLL. Most ABI improvement was achieved in former smokers who underwent DCB angioplasty. The later findings may have implications for future research.

## Electronic Supplementary Material

Below is the link to the electronic supplementary material.

Supplementary material 1 (DOCX 56 kb)
