# Supplementary material for: Multivariable Regression Analysis of Clinical Data from the Randomized-Controlled EffPac Trial: Efficacy of Femoropopliteal Drug-Coated Balloon Angioplasty
Source: Cardiovasc Intervent Radiol. 2020 Apr 1;43(6):840–9. doi: 10.1007/s00270-020-02452-2 (PMC7225207; doi:10.1007/s00270-020-02452-2)
Supplement: Supplementary file 1 — Supplementary material 1 (DOCX 56 kb) [file 270_2020_2452_MOESM1_ESM.docx]

**Online Resource 1**

**Multivariable Regression Analysis of Clinical Data from the Randomized-controlled EffPac Trial – Efficacy of Femoropopliteal Drug-Coated Balloon Angioplasty**

| **Table A1** Eligibility criteria |
| --- |
| **Inclusion criteria**   1. Age ≥ 18 years 2. Subject must agree to undergo the 6-month angiographic and clinical follow-up (at 12- and 24 months post-procedure) 3. Peripheral vascular disease Rutherford class 2-4 4. De novo stenotic/ re-stenotic lesion or occlusive lesions in the superficial femoral (SFA) and/or popliteal arteries (PA) 5. If the index lesion is re-stenotic, the prior PTA must have been >30 days prior to treatment in the current study 6. ≥70% diameter stenosis or occlusion 7. Target lesion length: ≤15 cm 8. Only one lesion per limb and per patient can be treated 9. ≥ one patent intrapopliteal run-off artery to the foot of the index limb 10. Successful endoluminal guidewire passage through the target lesion 11. Pre-dilatation prior to randomization 12. Life expectancy, in the investigators’ opinion of at least one year 13. Subject is able to verbally acknowledge and understand the aim of this trial and is willing and able to provide informed consent. |
| **Exclusion criteria**   1. Previous surgery in the target vessel 2. Patients who require a PTA balloon catheter in diameter size 4 mm or in diameter size greater 7 mm. 3. Major amputation in the same limb as the target lesion 4. Acute myocardial infarction within 30 days before intervention 5. Severely calcified target lesions in the SFA/PA resistant to PTA 6. Subjects requiring different treatment or raising serious safety concern regarding the procedure or the required medication 7. Women of childbearing potential except women with the following criteria:    1. post-menopausal (12 months natural amenorrhea or 6 months amenorrhea with serum FSH > 40mlU/ml)    2. sterilization after bilateral ovariectomy with or without hysterectomy    3. using an effective method of birth control for the duration of the trial: implants, injectables, combined oral contraceptives, intrauterine device (in place for a period of at least 2 months prior to screening) and with negative serum pregnancy test    4. sexual abstinence    5. vasectomy partner 8. Pregnant and nursing women 9. Acute thrombus aneurysm in the index limb or vessel 10. In-stent restenosis in the target lesion 11. Renal insufficiency with a serum creatinine >2.0 mg/dL at baseline 12. Platelet count <50 G/l or >600 G/l at baseline 13. Known hypersensitivity or contraindication to contrast agent that cannot be adequately pre-medicated 14. Subjects with known allergies against paclitaxel 15. Subjects with intolerance to antiplatelet, anticoagulant, or thrombolytic medications that would be administered during the trial 16. Dialysis or long-term immunosuppressant therapy 17. Current participation (or within the last 3 months) in another interventional study. |

**Definition of Study Endpoints**

LLL was defined as difference between minimum lumen diameter immediately after angioplasty and at 6-month follow-up. Restenosis occurred with > 50% diameter stenosis of the target lesion by angiography or peak systolic velocity ratio of ≥ 2.5 by duplex ultrasound, both adjudicated by core laboratory. Any clinically driven surgical or endovascular revascularization of the target lesion was considered TLR. Patients who underwent TLR within 12 months were included in the follow-up investigation of Rutherford-Becker category and ABI.

| **Table A2** Subgroup analysis with formal interaction testing regarding clinical, morphologic, and hemodynamic outcomes | | | | | | | | | | |
| --- | --- | --- | --- | --- | --- | --- | --- | --- | --- | --- |
| **Interaction terms**  **(variable* treatment)** | **Clinical** | | | | **Morphologic** | | | | **Hemodynamic** | |
|  | **TLR** | | **RB category** | | **LLL** | | **Restenosis** | | **ABI** | |
|  | Odds ratio  (95% CI) | P-value  (inter-action) | B coefficient  (95% CI) | P-value  (interaction) | B coefficient  (95% CI) | P-value  (inter-action) | Odds ratio  (95% CI) | P-value  (inter-action) | B coefficient  (95% CI) | P-value  (inter-action) |
| Age, years | 0.97 (0.06-16.48) | p=0.98 | 0.01 (-0.45-0.46) | p=0.98 | 0.20 (-0.36-0.76) | p=0.48 | 1.35 (0.52-3.49) | p=0.54 | -0.03 (-0.16-0.10) | p=0.66 |
| Sex  male vs. female | ^a^ |  | 0.09 (-0.65-0.84) | p=0.80 | -0.03 (-1.04-0.98) | p=0.95 | 0.86 (0.19-3.95) | p=0.85 | 0.01 (-0.20-0.22) | p=0.93 |
| Hypertension  yes vs. no | ^a^ |  | 0.20 (-0.80-1.20) | p=0.69 | -0.38 (-0.85-1.62 | p=0.54 | 0.22 (0.03-1.50) | p=0.12 | 0.04 (-0.25-0.32) | p=0.80 |
| Diabetes  yes vs. no | ^a^ |  | 0.00 (-0.73-0.73) | p=1.00 | -0.29 (-0.62-1.21) | p=0.53 | 0.51 (0.11-2.42) | p=0.39 | 0.01 (-0.19-0.22) | p=0.89 |
| Underweight  vs. normal w. | ^a^ |  |  | p=0.03 | -0.72 (-4.08-2.64) | p=0.77 | ^a^ | p=0.20 | ^a^ | p=0.45 |
| Pre obesity  vs. normal w. | ^a^ |  | -0.67 (-1.55-0.22) |  | -0.27 (-1.36-0.81) |  | 0.25 (0.04-1.74) |  | -0.10 (-0.35-0.15) |  |
| Obesity  vs. normal w. | ^a^ |  | 0.55 (-0.42-1.51) |  | 0.28 (-0.95-1.50) |  | 0.07 (0.01-0.92) |  | 0.07 (-0.21-0.35) |  |
| Current smoker  vs. non-smoker | ^a^ |  | -0.22 (-1.30-0.86) | p=0.44 | 0.84 (-0.59-2.27) | p=0.30 | 7.29 (0.80-66.47) | p=0.11 | 0.08 (-0.20-0.35) | p=0.02 |
| Former smoker  vs. non-smoker | ^a^ |  | 0.27 (-0.79-1.34) |  | 1.11 (-0.31-2.53) |  | 1.73 (0.16-19.05) |  | 0.33 (0.05-0.61) |  |
| ABI | 13.40 (0.01-26914.43) | p=0.50 | -1.23 (-2.87-0.42) | p=0.14 | -0.80 (-2.98-1.39) | p=0.47 | 0.27 (0.01-13.47) | p=0.51 | 0.24 (-0.10-0.58) | p=0.16 |
| RB category  2 vs. 4 | ^a^ | ^a^ | -0.36 (-1.20-0.47) | p=0.39 | -0.86 (-2.12-0.40) | p=0.18 | ^a^ |  | 0.08 (-0.17-0.33) | p=0.52 |
| RB category  3 vs. 4 | ^a^ |  |  |  | ^a^ |  | ^a^ |  | ^a^ |  |
| Lesion length | 0.61 (0.18-2.12) | p=0.44 | 0.03 (-0.06-0.12) | p=0.50 | -0.06 (-0.17-0.05) | p=0.26 | 0.98 (0.82-1.17) | p=0.84 | 0.01 (-0.02-0.03) | p=0.63 |

| **Table A2** Subgroup analysis with formal interaction testing regarding clinical, morphologic, and hemodynamic outcomes | | | | | | | | | | |
| --- | --- | --- | --- | --- | --- | --- | --- | --- | --- | --- |
| **Interaction terms**  **(variable* treatment)** | **Clinical** | | | | **Morphologic** | | | | **Hemodynamic** | |
|  | **TLR** | | **RB category** | | **LLL** | | **Restenosis** | | **ABI** | |
|  | Odds ratio  (95% CI) | P-value  (inter-action) | B coefficient  (95% CI) | P-value  (interaction) | B coefficient  (95% CI) | P-value  (inter-action) | Odds ratio  (95% CI) | P-value  (inter-action) | B coefficient  (95% CI) | P-value  (inter-action) |
| Calcification,  moderate vs. no | ^a^ |  | -0.20 (-0.92-0.53) | p=0.84 | 0.26 (-0.67-1.19) | p=0.58 | 0.76 (0.17-3.50) | p=0.94 | -0.14 (-0.34-0.06) | p=0.39 |
| Calcification, severe vs. no | ^a^ |  | 0.11 (-1.70-1.92) |  | 1.05 (-1.66-3.75) |  | ^a^ |  | -0.03 (-0.51-0.45) |  |
| Diameter stenosis, % | 0.20 (0.03-1.60) | p=0.13 | -0.30 (-0.66-0.07) | p=0.11 | -0.12 (-0.61-0.37) | p=0.64 | 1.44 (0.63-3.30) | p=0.39 | -0.02 (-0.13-0.09) | p=0.74 |
| Total occlusion  yes vs. no | ^a^ |  | -0.06 (-0.92-0.79) | p=0.89 | -1.09 (-2.24-0.06) | p=0.06 | 2.86 (0.55-14.90) | p=0.21 | 0.03 (-0.22-0.28) | p=0.83 |
| TASC B  B vs. A | ^a^ |  | -0.10 (-0.86-0.66) | p=0.79 | -0.73 (-1.69-0.23) | p=0.14 | 0.46 (0.10-2.09) | p=0.31 | -0.02 (-0.23-0.19) | p=0.84 |
| Dissection  yes vs. no | ^a^ |  | 0.02 (-0.71-0.74) | p=0.97 | 0.49 (-0.80-1.79) | p=0.65 | 0.54 (0.11-2.62) | p=0.45 | -0.09 (-0.29-0.12) | p=0.39 |
| Bailout stenting  yes vs. no | ^a^ |  | -0.16 (-1.17-0.86) | p=0.76 | -0.82 (-1.67-0.03) | p=0.45 | 1.68 (0.19-14.79) | p=0.64 | 0.10 (-0.19-0.39) | p=0.49 |

Interaction terms that were included into the final multivariable analysis were highlighted in grey

^a^ not applicable due to small number of events

*ABI* ankle-brachial index, *LLL* late lumen loss, *RB* Rutherford-Becker category, *TASC* inter-society consensus for the management of peripheral arterial disease classification, *TLR* target lesion revascularization

| **Table A3** Univariable analysis regarding clinical, morphologic, and hemodynamic outcomes | | | | | | | | | | |
| --- | --- | --- | --- | --- | --- | --- | --- | --- | --- | --- |
| **Variables** | **Clinical** | | | | **Morphologic** | | | | **Hemodynamic** | |
|  | **TLR** | | **RB category** | | **LLL** | | **Restenosis** | | **ABI** | |
|  | Odds ratio  (95% CI) | P-value | B coefficient  (95% CI) | P-value | B coefficient  (95% CI) | P-value | Odds ratio  (95% CI) | P-value | B coefficient  (95% CI) | P-value |
| Age, years | 0.69 (0.36-1.33) | p=0.27 | -0.03 (-0.26-0.19) | p=0.76 | -0.03 (-0.05-0.00) | p=0.05 | 0.70 (0.45-1.09) | p=0.11 | -0.02 (-0.09-0.04) | p=0.51 |
| Sex  male vs. female | 1.40 (0.40-4.87) | p=0.60 | -0.13 (-0.50-0.24) | p=0.50 | -0.04 (-0.54-0.47) | p=0.89 | 1.06 (0.50-2.25) | p=0.88 | 0.02 (-0.09-0.12) | p=0.77 |
| Hypertension  yes vs. no | 3.03 (0.36-25.30) | p=0.31 | -0.35 (-0.85-0.14) | p=0.16 | 0.56 (-0.05-1.18) | p=0.07 | 0.72 (0.27-1.91) | p=0.51 | -0.04 (-0.18-0.10) | p=0.57 |
| Diabetes  yes vs. no | 1.07 (0.34-3.35) | p=0.91 | -0.14 (-0.50-0.50) | p=0.45 | 0.25 (-0.21-0.70) | p=0.29 | 0.80 (0.38-1.68) | p=0.56 | -0.02 (-0.12-0.09) | p=0.78 |
| Underweight  vs. normal w. | 55.49 (1.88-1637.06) | p=0.10 | 0.42 (-1.14-1.98) | p=0.78 | 1.67 (0.01-3.34) | p=0.07 | 4.63 (0.35-61.23) | p=0.64 | -0.12 (-0.52-0.28) | p=0.41 |
| Pre obesity  vs. normal w. | 2.41 (0.46-12.55) |  | -0.12 (-0.56-0.32) |  | -0.30 (-0.83-0.24) |  | 0.94 (0.38-2.32) |  | -0.08 (-0.20-0.05) |  |
| Obesity  vs. normal w. | 1.09 (0.14-8.60) |  | -0.19 (-0.67-0.30) |  | 0.11 (-0.50-0.71) |  | 0.83 (0.30-2.34) |  | -0.11 (-0.25-0.03) |  |
| Current smoker  vs. non-smoker | 1.01 (0.23-4.52) | p=0.21 | 0.29 (-0.25-0.82) | p=0.23 | 0.17 (-0.55-0.88) | p=0.86 | 0.78 (0.28-2.13) | p=0.16 | -0.02 (-0.16-0.13) | p=0.22 |
| Former smoker  vs. non-smoker | 0.30 (0.05-1.71) |  | -0.04 (-0.57-0.49) |  | 0.07 (-0.64-0.77) |  | 0.41 (0.14-1.17) |  | -0.10 (-0.24-0.04) |  |
| ABI | 0.71 (0.04-12.85) | p=0.82 | -0.40 (-1.23-0.43) | p=0.34 | -0.79 (-1.85-0.27) | p=0.14 | 0.20 (0.03-1.37) | p=0.10 | -0.70 (-0.87-(-0.53)) | p<0.001 |
| RB category  2 vs. 4 | ^a^ |  | -0.25 (-1.68-1.18) | p<0.001 | 0.11 (-2.36-2.59) | p=0.98 | ^a^ |  | -0.09 (-0.49-0.31) | p=0.53 |
| RB category  3 vs. 4 | ^a^ |  | 0.82 (-0.57-2.21) |  | 0.05 (-2.36-2.47) |  | ^a^ |  | -0.02 (-0.41-0.37) |  |
| Lesion length | 0.99 (0.85-1.15) | p=0.86 | -0.06 (-0.10-(-0.01)) | p=0.01 | 0.00 (-0.01-0.00) | p=0.53 | 1.09 (0.99-1.19) | p=0.07 | 0.01 (0.00-0.02) | p=0.06 |

| **Table A3** Univariable analysis regarding clinical, morphologic, and hemodynamic outcomes | | | | | | | | | | |
| --- | --- | --- | --- | --- | --- | --- | --- | --- | --- | --- |
| **Variable** | **Clinical** | | | | **Morphologic** | | | | **Hemodynamic** | |
|  | **TLR** | | **RB category** | | **LLL** | | **Restenosis** | | **ABI** | |
|  | Odds ratio  (95% CI) | P-value | B coefficient  (95% CI) | P-value | B coefficient  (95% CI) | P-value | Odds ratio  (95% CI) | P-value | B coefficient  (95% CI) | P-value |
| Calcification,  moderate vs. no | 0.78 (0.25-2.42) | p=0.91 | -0.30 (-0.66-0.06) | p=0.04 | 0.24 (-0.22-0.70) | p=0.55 | 1.12 (0.53-2.36) | p=0.96 | 0.00 (-0.10-0.11) | p=0.08 |
| Calcification, severe vs no | ^a^ |  | -0.94 (-1.77-(-0.11) |  | -0.09 (-1.13-0.95) |  | 0.98 (0.22-4.48) |  | -0.25 (-0.48-(-0.03)) |  |
| Diameter stenosis, % | 1.84 (0.87-3.91) | p=0.11 | 0.38 (0.19-0.56) | p<0.001 | 0.00 (-0.03-0.02) | p=0.95 | 1.18 (0.79-1.76) | p=0.42 | 0.03 (-0.02-0.08) | p=0.28 |
| Total occlusion  yes vs. no | 2.42 (0.74-7.84) | p=0.14 | 0.50 (0.07-0.93) | p=0.02 | -0.19 (-0.75-0.75) | p=0.51 | 2.36 (1.04-5.35) | p=0.04 | 0.11 (-0.02-0.23) | p=0.10 |
| TASC  A vs. B | 2.13 (0.68-6.63) | p=0.19 | -0.09 (-0.46-0.29) | p=0.65 | 0.00 (-0.49-0.48) | p=0.99 | 2.00 (0.95-4.21) | p=0.07 | 0.12 (0.02-0.22) | p=0.03 |
| Dissection  yes vs. no | 0.75 (0.23-2.44) | p=0.64 | 0.18 (-0.18-0.54) | p=0.32 | 0.05 (-0.41-0.50) | p=0.84 | 0.71 (0.34-1.51) | p=0.38 | 0.05 (-0.05-0.15) | p=0.34 |
| Bailout stenting  yes vs. no | 0.88 (0.17-4.46) | p=0.87 | 0.46 (-0.04-0.96) | p=0.07 | -0.05 (-0.69-0.59) | p=0.88 | 0.65 (0.22-1.93) | p=0.44 | -0.10 (-0.24-0.04) | p=0.17 |

Variables that were included into the final multivariable analysis were highlighted in grey

^a^ not applicable due to small number of events

*ABI* ankle-brachial index, *LLL* late lumen loss, *RB* Rutherford-Becker category, *TASC* inter-society consensus for the management of peripheral arterial disease classification, *TLR* target lesion revascularization
